# Flow control in our vessels: vascular valves make sure there is no way back

**DOI:** 10.1007/s00018-012-1110-6

**Published:** 2012-08-25

**Authors:** Eleni Bazigou, Taija Makinen

**Affiliations:** 1 Lymphatic Development Laboratory, Cancer Research UK London Research Institute, 44 Lincoln’s Inn Fields, London, WC2A 3LY UK; 2 Present Address: Cardiovascular Mechanics Lab, Department of Bioengineering, Imperial College London, London, SW7 2AZ UK

**Keywords:** Valve, Lymphatic vessel, Vein, Endothelium, Morphogenesis

## Abstract

The efficient transport of blood and lymph relies on competent intraluminal valves that ensure unidirectional fluid flow through the vessels. In the lymphatic vessels, lack of luminal valves causes reflux of lymph and can lead to lymphedema, while dysfunction of venous valves is associated with venous hypertension, varicose veins, and thrombosis that can lead to edema and ulcerations. Despite their clinical importance, the mechanisms that regulate valve formation are poorly understood and have only recently begun to be characterized. Here, we discuss new findings regarding the development of venous and lymphatic valves that indicate the involvement of common molecular mechanisms in regulating valve formation in different vascular beds.

## Control of fluid flow through the vascular system: importance of valves

The circulatory system is composed of the heart and the blood vessels, which distribute nutrients, hormones, gases, and metabolic waste products in the body, and the lymphatic vessels that ensure that the extravasated fluid and proteins are drained from the tissues and transported back to the blood circulation. The beating of the heart generates pulsatile flow of blood that is pushed through a hierarchical network of arteries, capillaries, and veins, with heart valves controlling the flow of blood through the heart by opening and closing during the contractions of the heart. In addition, contraction of skeletal muscles compresses the walls of deep veins and drives the movement of blood inside the vessels, thus behaving like peripheral muscle pumps [1]. Additional mechanisms that activate the venous pumping system include arterial pulsation and the ankle joint pump, which is generated by ankle joint motion [2], while luminal valves in the veins ensure unidirectional blood flow (reviewed in [3]). Venous valves are required for efficient return of blood particularly from lower extremities, where the greatest gravitational forces are present.

Like the blood vasculature, the lymphatic system is composed of a hierarchy of vessels with specific features serving their unique functions: the blind-ended lymphatic capillaries (also called initial lymphatics) that absorb the interstitial fluid and the collecting lymphatic vessels that transport the lymph to the cardiovascular system (reviewed in [4]). In birds and amphibians, specialized lymph hearts are responsible for lymph propulsion while extrinsic mechanisms such as skeletal muscle contractions play a negligible role [5]. Mammals do not have lymph hearts and both intrinsic and extrinsic forces are important for efficient lymph propulsion. Extrinsic pump mechanisms include cardiac and arterial pulsations, skeletal muscle contractility, venous pressure fluctuations, peristaltic movement of the intestine, and respiration. The extrinsic mechanisms play an important role in controlling the opening of the specialized cell–cell junctions, so-called primary valves, in lymphatic capillaries [6, 7]. Collecting vessels are affected by the same extrinsic factors; however, their function is mainly controlled by intrinsic mechanisms that depend on the contraction of the smooth muscle cells around the vessels and the action of intraluminal valves that prevent backflow of the lymph [8, 9]. An important regulatory mechanism of lymphatic pumping is triggered by high nitric oxide (NO) concentration in the valve area, which is due to increased expression of eNOS in valve endothelia [10].

Consistent with their important functions, incompetence of venous valves has clinical implications and is associated with venous hypertension, orthostatic leg swelling, development of varicose veins, and leg ulcerations [3, 11, 12]. Dysfunction of lymphatic valves can similarly contribute to clinical disease. For example, lack of valves underlies lymphatic failure and consequent edema in patients with a form of primary hereditary lymphedema called Lymphedema Distichiasis [13, 14].

## Fluid dynamics of the valve

**Fig. 1:**
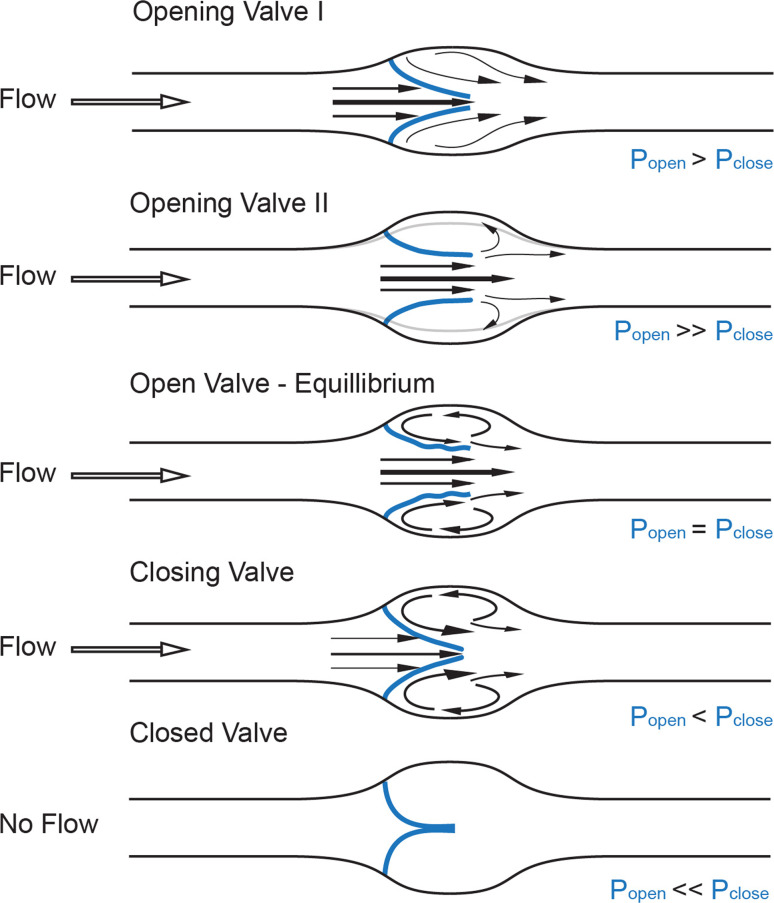
Fluid dynamics of a valve. Distinct phases of opening and closing of valve leaflets (*blue*): opening, equilibrium, closed, and closing, modified from [12]. Fluid pressure drop across the vessel drives direction of flow (*black hollow arrow*), while forces on either side of valve leaflets (*blue*,* P*_*open*_,* P*_*close*_) determine the leaflets’ position inside the lumen and the size of the valve orifice. *Black arrows* point at the distinct flow patterns as well as the fluid velocities at different parts of the valve pocket (relatively scaled to demonstrate magnitude differences), such as axial flow in the middle of the vessel and detached streamlines at the free ends of the open leaflets developing into vortices in the sinus. *Gray line* shows the level of the vessel distension during the opening phase of the valve

Recent advances in ultrasound technology have given insight into the physics of venous valve operation and have delineated the way the movement of the leaflets affects blood flow into four distinct phases of the valve cycle: opening, equilibrium, closing, and closed (Fig. 1) [15]. An increase in pressure behind the closed leaflets forces them to push back towards the vessel wall and create an opening while the stagnated blood between the leaflets and the sinus wall is pushed forward. Once open, the leaflets remain suspended in the flowing bloodstream and undergo self-excited oscillations [15], while the blood flow through the valve orifice separates at the oscillating edges of the valve leaflets to create one axial flow jet in the middle of the opening and two recirculation vortices of lower speed in the sinus, causing distention of the valve sinus [15, 16]. While the pressure in the recirculation vortex is in equilibrium with the pressure exerted on the inflow side of the leaflets, the valve remains open. Changes in any of these streams can shift this balance so that, with rising pressure on the outflow side and decreasing pressure on the luminal side, the valve leaflets simultaneously move towards the center of the vessel and then remain closed until a new cycle starts [15]. These hemodynamic events are predetermined by the solid mechanics of the valve leaflets and the perivalvular area, and constitute a self-sustained mechanism for competent valve operation [15, 17]. A similar flow separation model has been validated [16, 18, 19] at the orifice of the aortic valves of the heart, first proposed by Leonardo da Vinci [20], and was recently suggested to apply to lymphatic valves, as similar opening and closing motions of the lymphatic valve leaflets have recently been recorded in excised vessels and in vivo [21, 22].

## Valve morphogenesis

**Fig. 2:**
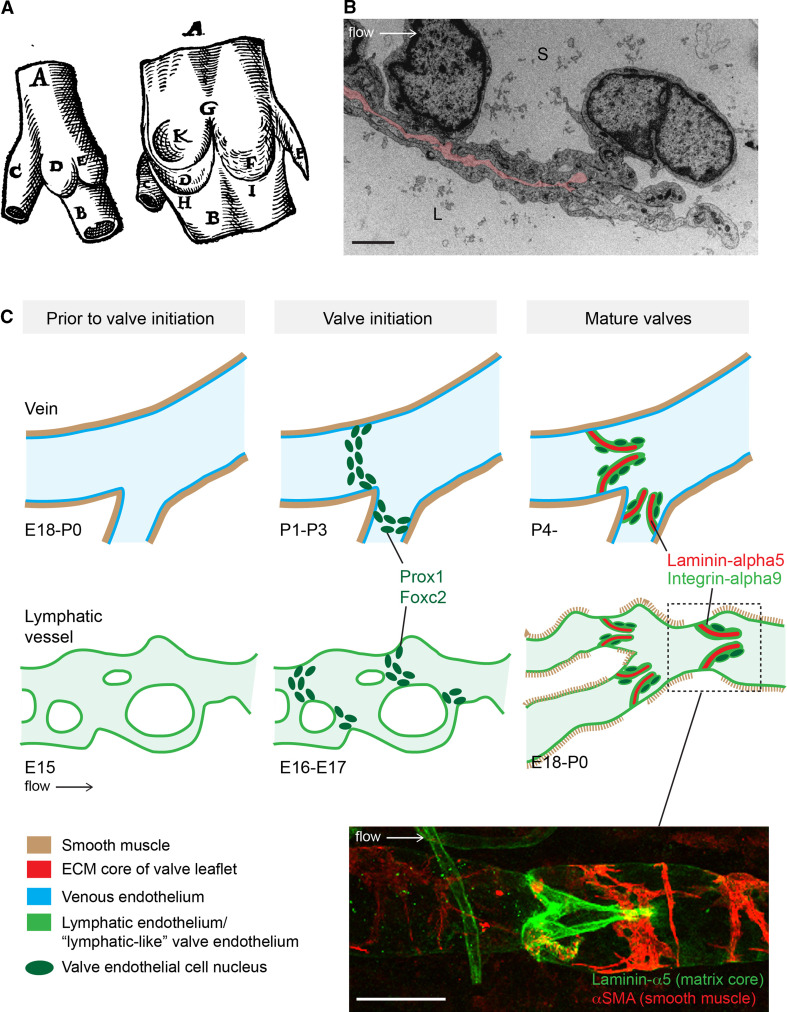
Similarities and differences between valve formation process in veins and lymphatic vessels. **a** First drawing of a valve, by Salomon Alberti in 1585 ([28], reproduced from [27], Copyright (1927), Royal Society Medicine Press, UK), showing the outside and inside of part of a leg vein (indicated by *A*, *B*) with a tributary vein (*C*). *D* and *E* indicate the two cusps of a bicuspid valve. **b** Transmission electron microscopy of the tip of a lymphatic valve leaflet *L* lumen, *S* sinus. Matrix core of the leaflet is highlighted in *pink* and flow direction is indicated by an *arrow*. *Scale bar* 2 μm. **c** Schematic of the developmental process of valve formation in veins (*top row*, *blue*) and lymphatic vessels (*bottom row*, *green*). Direction of blood/lymph flow and color codes representing different tissues are shown below. Developmental time-points, as determined for the valves in the proximal femoral vein and mesenteric lymphatic vessels in mice, are indicated below each stage; *E* embryonic, *P* postnatal. Note the presence of uniform smooth muscle coating (*brown*) in veins prior to valve initiation, while sparse coverage of SMCs is acquired to lymphatic vessels only after valve formation and concomitant remodeling of a primitive vascular plexus to mature collecting vessels. Valve initiation in both veins and lymphatic vessels is characterized by emergence of clusters of cells expressing high levels of Prox1 and Foxc2 transcription factors (*dark green* nuclei), predominantly near vessel branch points, and followed by formation of leaflets with two layers of endothelial cells expressing Integrin-α9 (*light green*) attached to Laminin-α5 positive matrix core (*red*). *Bottom* confocal micrograph of a dermal collecting lymphatic vessel stained for Laminin-α5 to visualize the extracellular matrix core of the valve leaflet (*green*) and αSMA to highlight smooth muscle cells around the vessel (*red*). *Scale bar* 50 μm

**Fig. 3:**
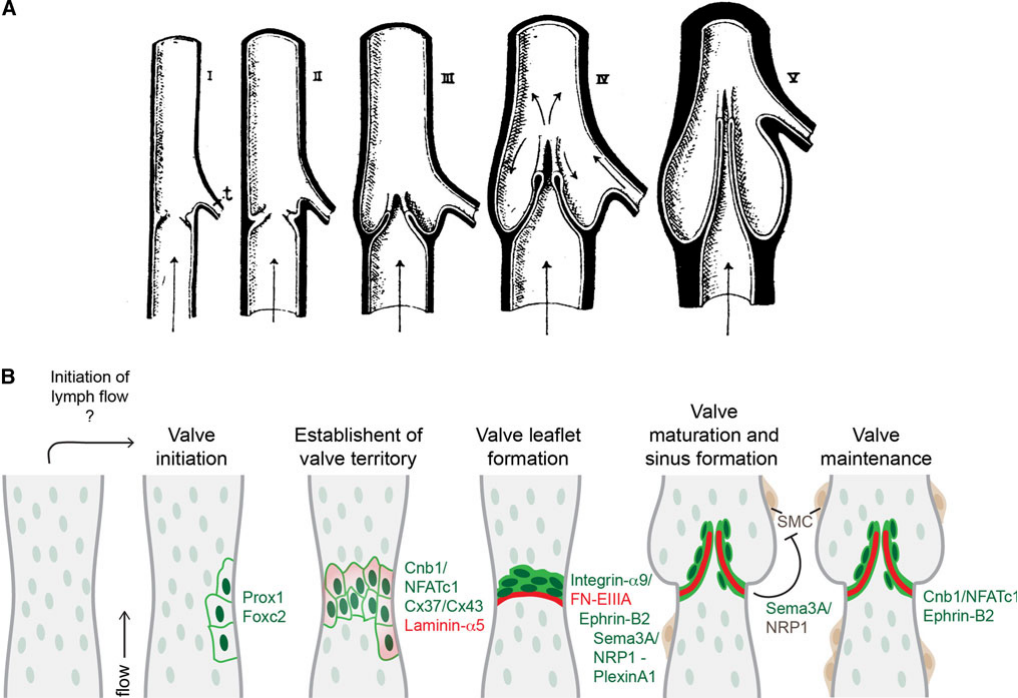
Model of valve morphogenesis. **a** Schematic representation of the development of a bicuspid venous valve by Kampmeier in 1927 ([28], reproduced from [27], Copyright (1927), Royal Society Medicine Press, UK). Endothelial layer is shown in *white* and mesenchymal layer in *black*. Flow direction is indicated by *arrows*;* t* tributary. **b** Schematic model of lymphatic valve morphogenesis. Some of the key regulators of different stages of valve formation are shown. *Green text* indicates expression in lymphatic endothelial cells, *dark brown* smooth muscle cells, and* red* extracellular matrix components. The initiation of valve formation coincides with the initiation of lymph flow in mesenteric lymphatic vessels. Clusters of endothelial cells expressing high levels of Prox1 and Foxc2 transcription factors (*dark green* nuclei) emerge at the sites of developing valves, which is followed by deposition of matrix molecules, such as Laminin-α5 (*red*) and establishment of valve territory via Calcineurin and Connexin signaling. Valve leaflet formation is initiated by the formation of an endothelial cell ring-like constriction and depends on Integrin-α9-mediated assembly of FN-EIIIA matrix. Repulsive signaling between Sema3A and NRP1 maintains valve areas free of smooth muscle cells (*SMC*, *brown*), while Calcineurin and Ephrin-B2 signaling regulate the maintenance of valve leaflets

The mature venous and lymphatic valves are typically bicuspid, and are composed of two luminal leaflets that consist of two layers of endothelial cells separated by a defined connective tissue core ([23–26]; Fig. 2a, b). Unicuspid venous valves, as well as bicuspid, and at least in veins, tricuspid valves composed of leaflets of unequal size have also been observed [23, 27]. The development of venous [28] and lymphatic [29] valves was first characterized by Kampmeier in the late 1920s (Fig. 3a):

“The bicuspid valve begins as a pair of inconspicuous and more or less transversely placed endothelial ridges inside the vein. By the invasion of the mesenchyme, the elongation and slanting of the ridges, and the formation of a concavity on their leeward face, the anlagen are gradually converted into vulvar sacs. Coincident with such changes, the venous cavity flares outward at the level of these sacs, so aiding in the production of their sinus [28].”

Recent studies using high-resolution imaging techniques have confirmed Kampmeier’s accurate description of the basic morphogenic events during valve formation. In addition, analyses of genetic mouse models have provided important insight into the molecular and cellular mechanisms regulating morphogenesis of both lymphatic and venous valves. Below, we describe the sequence of developmental events and the molecular mechanisms operating during early onset of valve development, leaflet elongation, and valve maintenance in lymphatic vessels and veins.

### Onset of valve development: defining the positions

While it is not fully understood how the process of valve development is initiated, it was recently shown that mechanical forces caused by fluid flow regulate the expression of key molecular regulators and cellular behaviours associated with valve-forming lymphatic endothelial cells [22]. Mechanical stimulus from fluid flow may therefore establish the locations where lymphatic valves develop. Indeed, valve position is not stochastically determined: although venous valves may follow each other in immediate succession, more frequently they are observed at stereotypic positions distal to the entrance of tributaries [27]. Similarly, lymphatic valves are often found in vessel bifurcations and branch points [22, 24], i.e. in geometries that are characterized by distinct flow and shear stress patterns [30]. Lymphatic endothelial cells that were exposed to oscillatory fluid shear stress, which was used to model the disturbed flow present in vessel branches [30], were shown to recapitulate multiple aspects of endothelial cell responses observed during early stages of valve formation, including upregulation of Foxc2 and activation of NFAT/Calcineurin signaling, as well as cytoskeletal rearrangement and changes of cell shape and alignment [22]. Notably, however, the timing of the onset of valve development appears to be different in veins and lymphatic vessels (Fig. 2c). In the mesenteric lymphatic vessels in mouse, the initiation of valve formation is correlated with the onset of lymph flow [22] and occurs concomitantly with remodeling of a primary lymphatic vascular plexus, characterized by a branched network of capillary-type vessels, into functional collecting vessels, while no de novo formation of luminal valves has been reported in established mature vessels ([24, 31], Fig. 2c). In contrast, the development of valves in the femoral vein in mouse was shown to occur during early postnatal development [32], indicating that the process starts after flow has already been present in the vessels and the gross morphology of the vein is no longer changing. However, in human fetuses, venous valves were already observed during early development (at 13 weeks of pregnancy) [33], and, as in the lymphatic system, it appears that venous valve initiation in some vascular beds occurs concomitantly with the remodeling of a venous plexus into hierarchy of deep, superficial and communicating veins [34]. Apart from experiencing different shear forces generated by the friction of the flowing blood or lymph on the surface of the vessels, the tangential wall tension caused by fluid pressure in the veins and lymphatic vessels is likely to be different. Venous valve development starts after the vessels have acquired a uniform smooth muscle cell coverage, which controls lumen shape and size and prevents overdistension. In contrast, the initial stages of lymphatic valve development precede smooth muscle cell recruitment to the vessels ([24, 31], Fig. 2c). These observations suggest that endothelial cells that are found at valve initiation sites in veins and lymphatic vessels experience very different levels of shear stress and tangential wall tension, yet they undergo similar molecular and morphological changes (see below), suggesting that factors other than flow are additionally involved in the initiation of valve development.

Interestingly, the early stages of heart valve formation share some common features with lymphatic and venous valve development. In mice, heart valve formation occurs as a result of endocardial cushion formation when signals derived from myocardium induce an endothelial-to-mesenchymal transition of adjacent endothelial cells (reviewed in [35, 36]). These endothelial-derived progenitors contribute to valvuloseptal structures and valve interstitial cells that play an important role in the remodeling of the valve extracellular matrix and formation of the leaflets [35, 36]. In zebrafish, however, heart valves appear to emerge at the atrioventricular boundary directly through an invagination of the endothelium, rather than from mesenchymal cushion [37], similarly as described for venous and lymphatic valves. In addition, like lymphatic valve morphogenesis [22], the early stages of zebrafish heart valve development are characterized by transition of the valve-forming cells to cuboidal shape [38]. Importantly, intracardiac hemodynamics were also shown to play a critical role in heart valve development: oscillatory blood flow between the atrium and ventricle was found to lead to upregulation of the shear-responsive gene Klf2a, an essential hemodynamic regulator gene ([39], Klf2 in mice), and important regulator of normal valvulogenesis [40]. Interestingly, this study suggested that the effects of blood flow operate only after an initial patterning that is guided by a genetic program, since the early stages of valve development, characterized by clustering of endothelial cells at the atrioventricular boundary and formation of an endothelial ring, did not require flow, and nor did they depend on Klf2a function [40]. Finally, some of the molecular mechanisms regulating the formation of valves in different parts of the vascular system are shared, such as the requirement of Calcineurin signaling for heart and lymphatic valve development ([22, 31, 41, 42], see below).

### Establishment of the valve territory

The first indication of lymphatic valve development is the appearance and clustering of cells expressing elevated levels of two transcription factors, Prox1 and Foxc2, in defined positions along the vessel ([22, 24, 31, 32]; Figs. 2c, 3b). This population of valve-forming endothelial cells subsequently rearranges and reorients perpendicular to the longitudinal axis of the vessel to form a ring-like constriction ([24, 32]; Fig. 3b). Cell rearrangement coincides with the deposition of extracellular matrix containing Fibronectin-EIIIA/EDA (FN-EIIIA) splice isoform and Laminin-α5, and upregulation of the cell–matrix adhesion receptor Integrin-α9 in valve-forming endothelial cells ([24]; Fig. 3b). Compared with other endothelial cells on the vessel wall, the valve-forming cells also express higher levels of certain junctional molecules such as CD31/PECAM-1, VE-Cadherin, and specific Connexins, while the expression of transmembrane proteins LYVE-1 and NRP2 is downregulated [22, 43]. The valve-forming cells can therefore be defined as a molecularly distinct population of lymphatic endothelial cells. Similarly, in the veins, one of the first signs of valve development is the induction of Prox1 expression, which is followed by upregulation of Integrin-α9 [32]. In addition, the transmembrane EphB ligand Ephrin-B2 is upregulated in the valve-forming venous endothelial cells during early stages of valve development. Prox1 was also found in endothelial cells of lympho-venous and cardiac valves [44, 45]. Intriguingly, previous studies have established Prox1 as a master regulator of lymphatic endothelial cell fate [46], while Ephrin-B2 is a well-established marker of arterial and lymphatic endothelia [47, 48]. The unexpected expression of these genes in developing venous valves suggests that the valve endothelial cells possess a unique identity. Interestingly, the venous endothelial cells, considered to represent terminally differentiated cell types, are thus capable of switching their identity upon initiation of valve formation.

Upon their induction, Prox1 and Foxc2 control, in response to flow, the expression and activation of the two important downstream regulators of valve formation, Connexin-37 and NFATc1 [22, 31, 43], suggesting their key role in further defining the identity of valve-forming cells. In agreement, *Foxc2* deficiency in mice and humans leads to lymphatic valvular aplasia [14, 31]. Consequently, human patients with *FOXC2* mutations develop lymphedema [49]. Reflux in great saphenous veins in these patients suggests an important function for FOXC2 also in the formation of venous valves [13]. While the function of Prox1 in venous or lymphatic valves has not been investigated, *Prox1* deficiency in mice leads to defective lympho-venous valve formation [45], implying that Prox1 is similarly involved in controlling valve development in different vessel types. Interestingly, Gata2, another transcription factor that shows a prominent expression in lymphatic valves, was found to regulate the expression of Prox1, Integrin-α9, and Ephrin-B2, suggesting that it may also play an important role in early stages of valve development [50]. Inactivating mutations in *GATA2* were identified as responsible for Emberger syndrome, an autosomal dominant primary lymphedema that is associated with a predisposition to acute myeloid leukemia (AML) [51], as well as in familial myelodysplastic syndrome/AML or MonoMAC syndrome and primary lymphedema [50]. However, it is not known whether valve defects underlie the lymphatic failure in these patients.

Connexin-37 (Cx37) is highly expressed in lymphatic valve-forming cells and regulates their rearrangement to a ring-like constriction during early stages of valve development, suggesting the importance of gap-junctional communication during this process ([22, 43]; Fig. 3b). Two other Connexins, Cx43 and Cx47, were shown to be enriched in mature lymphatic valves [22, 43]. Interestingly, Cx43 deficiency in mice also led to abnormal lymphatic development, including defective valve formation [43], while *GJC2* (encoding Cx47) was found to be mutated in lymphedema patients [52, 53]. These studies suggest a more general role for gap-junction-mediated signaling in the lymphatic vasculature. Calcineurin signaling, which regulates nuclear translocation of NFAT transcription factors, is also activated during early stages of lymphatic valve development and is required for the formation of a defined boundary of valve-forming cells [22, 31].

### Development of valve leaflets and sinus formation

The development of valve leaflets is initiated by the formation of a transverse ridge on the vessel wall, which is followed by the elongation of the leaflets into the vessel lumen and formation of the commissures ([32]; Fig. 3a, b). In both lymphatic vessels and veins, this process critically depends on the assembly of an extracellular matrix core within developing leaflets through interaction of a cell–matrix adhesion receptor integrin-α9 with one of its ligands, Fibronectin, containing the alternatively spliced EIIIA/EDA domain [24, 32]. Interestingly, FN-EIIIA is deposited specifically at the sites of developing lymphatic valves and is subsequently localized at the free ends of the extending leaflets, suggesting requirement for an integrin-specific mechanism of fibronectin fibrillogenesis in the formation of the valve matrix core [24].

Other signaling pathways important for both lymphatic and venous valve formation include Ephrin-B2, a transmembrane ligand for EphB receptor tyrosine kinases [32, 48]. In particular, ‘reverse signaling’, mediated via binding of PDZ domain containing effector molecules to the cytoplasmic domain of Ephrin-B2, was shown to be critical for normal lymphatic valve formation [48]. However, the precise morphogenetic process affected by loss of Ephrin-B2 and the critical receptor(s) mediating this effect have not been characterized. Interestingly, apart from its interaction with EphB receptors, Ephrin-B2 regulates the internalization and signaling of the major lymphangiogenic receptor tyrosine kinase, VEGFR-3 [54], which is strongly expressed in the developing and mature lymphatic valves [31], and transiently in the developing venous valves [32]. The function of VEGFR-3 in valve formation has not been directly studied; however, VEGFR-3 signaling was found to co-operate with Foxc2 in the development of lymphatic valves [14]. In addition, Milroy patients who present with a congenital form of lymphedema caused by inactivating *VEGFR3* mutations have reflux in their great saphenous veins [55], pointing to a potential role for VEGFR-3 in valve development.

High expression of another endothelial receptor tyrosine kinase, Tie2, was reported in venous valves, starting from the earliest developmental stages [32]. Interestingly, Angiopoietin2, the ligand for Tie2, is required for the maturation of the lymphatic vessels and the formation of lymphatic valves [56]. Experiments using cultured endothelial monolayers in vitro revealed that Tie2 is upregulated and activated upon exposure of cells to shear stress [57, 58], suggesting that Tie2 mediated signaling may be involved in regulating flow-mediated responses, and thus early stages of valve formation. Similarly, shear stress induces the activation of the phosphoinositide 3-kinase (PI3K)/Akt pathway [59], which is activated under exposure to flow downstream of Tie2 signaling [57, 58]. While the function of Tie2 in valve formation has not been studied, it is interesting to note that mice lacking the regulatory isoforms of the class Ia PI3K, p85α/p55α/p50α, show severe defects in the intestinal lymphatic vessels, including lack of valves [60]. In addition, lack of Akt1 leads to more specific defects in the formation of lymphatic valves in the superficial collecting vessels of the skin [61].

Concomitant with the formation of the valve leaflets, the vessel wall opposite the leaflets dilates to create a pocket on the side of each leaflet called the valve sinus. It has been demonstrated that the endothelial cells in the venous valve sinus behave differently from the ones in avalvular areas and have the ability to stretch twice as much [62]. Similar measurements have not been made in lymphatic valve sinuses; however, it is known that they are devoid of smooth muscle cells [14], which is likely to enable more extensive stretching of the vessel wall in this region. The importance of the lack of smooth muscle cells in lymphatic valves for their normal development and function was recently demonstrated. Sema3A, produced by lymphatic endothelial cells, was shown to repel smooth muscle cells expressing its receptor, NRP-1, thereby maintaining valve areas smooth muscle free [63, 64]. Sema3A was additionally shown to regulate valve leaflet formation via interaction with NRP-1 and PlexinA1 on valve endothelial cells [63]. Other molecular regulators that may play a role in valve sinus formation or function in veins include anticoagulant markers endothelial protein receptor (EPCR) and thrombomodulin that are strongly expressed on venous valve leaflet and sinus, while procoagulant factors, such as VWF, present the opposite pattern [65, 66]. Such distribution of anti- and procoagulant factors in the valve pocket favors anticoagulation, and thus is protective from thrombosis.

### Establishment of the unique cellular identity of valve endothelium

**Fig. 4:**
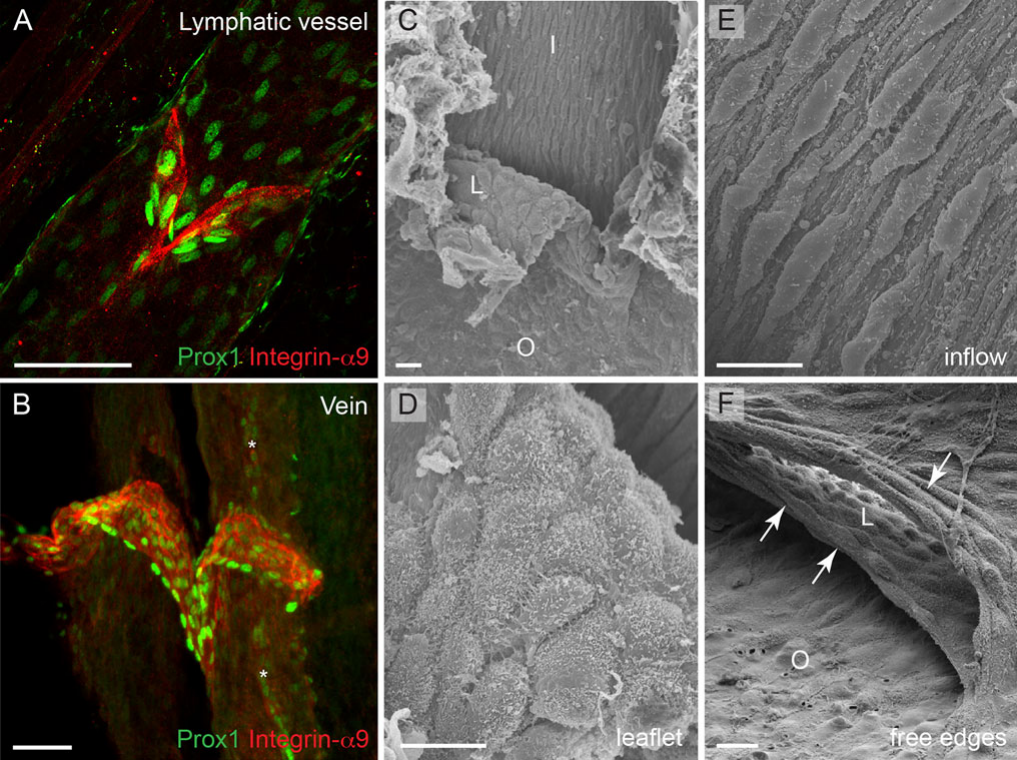
The molecular identity and morphology of valve endothelial cells. **a**, **b** Confocal micrographs of a mesenteric lymphatic vessel (**a**) and a femoral vein (**b**) stained for Integrin-α9 and Prox1 to visualize valve endothelial cells (*red*) and their nuclei (*green*), respectively. Image (**b**) reproduced from [32]), Copyright (2011), The Journal of Clinical Investigation, USA). *Asterisks* in (**b**) indicate a Prox1+ lymphatic vessel running in parallel of the vein. **c**–**f** Scanning electron microscope micrographs of a venous valve, showing different endothelial cell phenotypes (regarding cell shape and alignment) in different parts of the valve. Cells on the leaflet show rounded morphology (**d**) while cells on the inflow side (upstream) of the valve show elongated morphology and align in flow direction (**e**). The free edges of the leaflets are composed of cells that show transverse orientation and are highly elongated (**f**, *arrows*). *I* inflow, *O* outflow, *L* leaflet. *Scale bars* (**a**, **b**) 50 μm, (**c**, **f**) 10 μm, (**d**, **e**) 5 μm

The shared molecular identity and genetic regulation of valves in veins and lymphatic vessels, characterized by expression of several genes previously thought to be restricted to lymphatic and/or arterial endothelium such as Foxc2, Prox1, Integrin-α9 and Ephrin-B2 (Fig. 4a, b), raises the interesting question of how the unique valve endothelial cell identity is established. As discussed above, the initiation of lymph flow and the resulting shear forces were suggested to define the positioning, but also the identity, of valve-forming cells [22]. This study reported upregulation of Foxc2 in vitro in lymphatic endothelial cells that were subjected to oscillatory flow, while Prox1 levels were not affected. In contrast, in another study, flow-induced downregulation of Prox1 was observed both in cultured cells in vitro and in adult lymphatic vessels in vivo [67]. In addition, Prox1 was upregulated in embryonic mesenteric lymphatic vessels that were cultured ex vivo in the absence of fluid flow [22], which could be regarded as evidence for flow-induced downregulation of Prox1 expression that normally occurs in lymphangions (lymphatic vessel segments inbetween two consecutive valves). It should be noted that the first study examined flow-regulated responses under oscillatory shear stress, which was used to mimic disturbed flow patterns in the immature lymphatic vascular plexus, while the latter study used steady laminar flow. In addition, the shear stress levels that were applied were different. Since no measurements of shear or tangential wall stresses that are present in the developing lymphatic vessels are available, future work should investigate in more detail the precise mechanisms of valve initiation by lymph flow.

While the onset of blood flow cannot provide a complete explanation for the timing of the initiation of venous valve formation that occurs postnatally, the observed molecular and morphological differences between endothelial cells in different parts of the valve suggest the involvement of flow-induced forces in modulating cellular phenotypes during later stages of valve formation. For example, cells that are located upstream of the valve are elongated and aligned in the direction of flow, while the cells on either surface of the leaflets or lining the juxtaposed vein wall (valve sinus) appear to have a rounded or cuboidal morphology ([32]; Fig. 4c, f). Another study reported differences in the shape of cells located within different regions of the leaflet; endothelial cells on the medial surface of the valve leaflet were found to orient parallel to the long axis of the vessel, whereas those on the lateral surface aligned perpendicular to that axis [68]. Interestingly, the free ends of the valve leaflets are lined by endothelial cells that have a transverse arrangement and fusiform morphology (Fig. 4f). Lymphatic valve endothelial cells acquire a similar morphology in the respective areas of the valve; for example, appearing fusiform on the free edges [69] and elongated upstream of the valve [22, 24]. The elongated morphology of endothelial cells in venous and lymphatic vessel segments devoid of valves corroborates the observed steady attached flow conditions the cells experience, while the cuboidal shape of cells lining the leaflets suggests unsteady or oscillating shear. According to Poiseuille’s equation that describes parabolic velocity profile for laminar flow inside the tube, valve endothelial cells located in the most stenotic regions of the vessels, i.e. on the leaflet, experience higher wall shear stress than the cells lining the vessel wall; however, it is unlikely that this is a steady condition. Instead, valve endothelial cells are likely to experience unsteady flow patterns due to the oscillatory movement of the leaflets during equilibrium phase as well as small volume regurgitation [70], which is reflected in their cuboidal morphology. At the molecular level, differential expression of Prox1 and Ephrin-B2 was observed in vivo in cells that are exposed to different flow conditions within the valve. While Prox1 was predominantly expressed in the fusiform cells on the free edges of the valve leaflets (Fig. 4b), Ephrin-B2 was present on the leaflet and on the sinus wall on the outflow side of venous valve [32]. Intriguingly, the Prox1+ fusiform cells are highly elongated [32], which is in contrast to the role of Prox1 in regulating transition to a cuboidal cell shape during early stages of lymphatic valve development [22]. In summary, these observations suggest that the morphological and molecular characteristics that valve endothelial cells acquire reflect the distinct forces they experience. Consistent with this, differences in flow patterns and shear stress magnitude were previously shown to regulate gene expression and arterial-venous [71, 72] as well as lymphatic differentiation [67]. However, in addition to different forces, the cells experience different extracellular environments, with specific matrix components, such as Laminin-α5, present in the valve leaflet compared to the vessel wall, and lack of smooth muscle cell contact in valve endothelia. These factors are also likely to play an important role in defining the cellular identity of valve endothelial cells.

## Valve maintenance in adults and during aging

The mature venous valve leaflets are thicker at the sites of attachment to the vessel wall containing condensely packed collagen fibers; however, this deteriorates with age, thus making the valve leaflets more fragile [73]. It has also been recognized that stagnation of blood flow at the junction of valve leaflet and vessel wall can initiate microthrombus formation (reviewed in [17]). Although venous valves are designed to minimize stagnation of circulating blood, secondary vortices developing deep in the pockets of the valve leaflets can create potential stagnation zones. Such regions of unsteady flow and low velocities could cause the activation of platelets that together with red blood cells can form aggregates followed by infiltration of white blood cells through the vessel wall and initiation of responses similar to those observed in areas of trauma or atheroprone regions, while NFAT in blood vascular endothelia is regulating the activation of proinflammatory cells [74–76]. In addition, the naturally hypoxic environment inside the veins can further induce inflammatory responses in endothelial cells found in stagnation zones, by inducing generation of reactive oxygen species and NFκB [15, 17]. With ageing, a less thromboresistant phenotype present in the venous valve and possible alterations in the shear stress pattern or magnitude due to inflammation can lead to valvular lesions that can have important consequences on the normal function of the vasculature [77]. In addition, age-related fibrosis and thickening of venous valve leaflets and their decreased compliance can disrupt normal blood flow and affect the duration of blood stasis in the valve sinus, which can further abrogate the development of thrombus [78, 79].

At the molecular level, little is known about the mechanisms regulating valve maintenance. Studies on genetic mouse models have indicated important functions for both Integrin-α9 and Ephrin-B2 in the maintenance of venous valves, since conditional ablation of either gene in adult mice led to regression of valve leaflets [32]. Ephrin-B2 is additionally required for lymphatic valve maintenance [32]. In addition, loss of *Cnb1* in neonatal mice led to the regression of lymphatic valve leaflets in mice, indicating a continuous requirement for Calcineurin signaling for valve maintenance [22].

## Final remarks

The first observations on the development of lymphatic and venous valves were made nearly 100 years ago [28, 29], and today their importance for maintaining vascular function is well recognized, yet only recently have the molecular mechanisms that regulate valve formation begun to be elucidated. High-resolution imaging techniques and genetic mouse models that allow targeting and visualization of venous and lymphatic valves, as well as modeling of human valve pathologies, will continue to increase our understanding of the mechanisms that regulate valve morphogenesis and contribute to diseases associated with or caused by valvular dysplasia. Important questions of clinical relevance that still remain to be investigated in more detail are how valve development is initiated and what are the mechanisms that regulate the maintenance and normal function of valves during adulthood and aging. Finally, identification of shared and distinct pathways that regulate different types of valves should enable development of diagnostic and therapeutic approaches for specific targeting of venous and lymphatic valves.
